# The social marketing paradox: challenges and opportunities for the discipline

**DOI:** 10.1007/s12208-021-00308-0

**Published:** 2021-08-22

**Authors:** M. Bilal Akbar, Liz Foote, Alison Lawson, Jeff French, Sameer Deshpande, Nancy R. Lee

**Affiliations:** 1 Nottingham Trent University, Nottingham, UK; 2 Antioch University New England, NH Keene, USA; 3 University of Derby, Derby, UK; 4 Strategic Social Marketing Ltd, London, UK; 5 Griffith University, Griffith, Queensland Australia; 6 Social Marketing Services, Seattle, WA USA

**Keywords:** Social marketing, Challenges, Opportunities, Branding, Competition, Reach

## Abstract

This paper contributes to emerging discourse about the ongoing challenges and opportunities of social marketing as a discipline. The paper presents a qualitative perspective on existing challenges faced by social marketing and offers suggestions for addressing these challenges. Nine semi-structured interviews with social marketing academics and practitioners from six different countries were conducted. Thematic analysis was used to analyse and interpret the qualitative data. The study provides insight into existing challenges for social marketing, classified into three key themes according to their position within or outside of the discipline: 1) poor branding of the discipline as an internal challenge, 2) competing disciplines as an external challenge, and 3) overall reach of the discipline, seen as both an internal and external challenge. The findings suggest that social marketing needs to overcome poor branding issues to sufficiently address external challenges. We conclude by arguing for a more robust marketing of the discipline. While scholars have identified the challenges and opportunities for social marketing as a discipline, they have paid little attention to examining these challenges from the viewpoint of expert practitioners and academics. This paper presents a nuanced contextual understanding of the identified challenges through a qualitative perspective and explores how social marketing can overcome these challenges.

## Introduction

Social marketing marks its fiftieth anniversary in 2021, drawing much celebration amongst an ever-growing cadre of academics and practitioners who have embraced social marketing to drive social and environmental change. Kotler and Zaltman (1971) defined social marketing in their seminal paper as *"the design, implementation and control of programs calculated to influence the acceptability of social ideas and involving considerations of product planning, pricing, communication, distribution and marketing research"* (p5). To date, social marketing has made its most significant strides within the domain of public health, among the many disciplines that have used and recognised it (Deshpande, 2019; Kassirer et al., 2019; Kotler, 2011). It has been noted that "*the discipline has reached a new level of organisation and critical mass as it has grown and matured beyond its adolescence and early adulthood phase"* (Kassirer et al., 2019, p.209). However, the discipline itself is not without its challenges. Over the decades, the social marketing discourse has identified, described, and debated several issues surrounding the discipline's distinctiveness and acceptance (Deshpande, 2019). While some shifts have occurred through the years in the topics of discourse (Lee, 2020), many of the same issues continue to be critically discussed without an apparent resolution. Thus, the social marketing discipline still faces challenges that must be addressed to continue its advancement. We contend that some of these challenges should be elevated to the status of a 'threat", meaning that if these challenges are not sufficiently addressed, the discipline will not only fail to advance but could face setbacks.

Additionally, the language of social marketing in theory and practice warrants attention, as *"a deviation is noted from social marketing rhetoric in the interpretation of exactly what constitutes social marketing"* (Akbar et al., 2021, p.12). Finally, we believe that there remains a diverse and nuanced array of perspectives that have yet to be sufficiently captured. Therefore, we explore the current landscape of social marketing's challenges and opportunities, addressing the following research question:

- What are the current challenges and opportunities faced by the discipline of social marketing?

We have chosen a qualitative approach using semi-structured interviews as this method lends itself to deeper insights gained through a rich description (Creswell, 2007; Patten & Patten, 2018). We amplify the voices of social marketing thought leaders (academics and practitioners) in the hope of articulating the urgency faced by the discipline. This study contributes to the knowledge base by presenting a qualitative perspective on persistent social marketing challenges and potential strategies to safeguard and advance the discipline. We offer several solutions to address "wicked problems" (Churchman, 1967) that continue to plague humanity and our natural environment.

## Literature review

The earliest criticism of social marketing came from those who objected to broadening the marketing concept (Carman, 1973; Luck, 1969). While the discipline has undeniably moved beyond this, several critiques remain linked to the discipline's ongoing challenges, as discussed below.

### Individual-level vs broad approach

We assert that despite its maturity as a discipline, social marketing is still not widely known. For example, Andreasen (2002) characterised social marketing as having *"poor brand positioning"* (p.4), an assessment that continued to be echoed a decade later (Beall et al., 2012) and through to today (Kassirer et al., 2019; Lee, 2020). As a result, much confusion and misunderstanding exists about social marketing and what it is not (French & Gordon, 2020; McDermott et al., 2005; Wood, 2012). Some of the confusion can be attributed to early and evolving definitions of social marketing (Andreasen, 2006; Dibb & Carrigan, 2013), which gave the impression that social marketing was solely focused on behaviour change only at the individual level. Beginning in the 1990s, many began to criticise this "downstream" focus as too narrow and called for social marketing to broaden its approach and adopt more midstream and upstream methods to address policy and decision-maker audiences along with structural factors (Andreasen, 1995, 2010; Gordon, 2013; Hastings & Donovan, 2002).

While many scholars have adopted systems-level approaches (Brennan et al., 2016; Brychkov & Domegan, 2017; Domegan et al., 2016; Kennedy & Parsons, 2012; Kennedy et al., 2018; Lee & Kotler, 2019; Trenchard-Mabere, 2016), individual-level focus still dominates (Dibb & Carrigan, 2013; French & Gordon, 2015; Truong et al., 2019). Additionally, Kotler observed that social marketing has been *"neglected at the midstream and upstream levels"* (Dibb & Carrigan, 2013, p.1381). However, as noted in editorials following the 2015 World Social Marketing Conference (Gordon et al., 2016) and the 2019 World Social Marketing Conference (Veríssimo, 2020), an uptick in presentations that featured behaviour change efforts beyond the level of the individual was an encouraging sign for the discipline. This positive shift is ongoing, but we maintain that a more concerted effort is needed to address this issue. This would be timely because social marketing currently intends to contribute to the United Nations 2030 Agenda for Sustainable Development to engage, connect, and partner with other stakeholder groups to address complex and upstream level issues.

### Social marketing vs social media

Confusion abounds with the similarly named "social media". Lee and Kotler (2019) recounted the origin of the name "social marketing", chosen as a shortened term for "social cause marketing". However, just as social marketing was gaining traction within the realm of public health in the 1990s, a new form of communications emerged, consisting of various individual online networking platforms collectively known as "social media". Furthermore, Lee and Kotler (2019, p.51) noted, *"little did we know that social marketing would later be confused with 'social media marketing' where some practitioners of social media marketing today shorten their phrase to 'social marketing'".* Indeed, any online search for "social marketing" invariably returns responses primarily related to social media. This situation is seen as serious enough to be deemed not just a challenge but a threat to the discipline, as noted in a SWOT analysis of social marketing (Deshpande, 2019). While we concur that this issue should be considered a threat, we have also pointed out that a diversity of viewpoints exists within the social marketing community (Lee, 2020) via ongoing dialogue in professional and academic communities–regarding the nature and degree of this "threat". Therefore, some form of consensus on the role of social media in the confusion about social marketing is needed to be best discussed and addressed by the discipline.

### Social marketing vs marketing

The term "social marketing" has also been seen as problematic due to ongoing perceptions about commercial marketing (Andreasen, 2006; Deshpande, 2019; Hastings & Domegan, 2014; Lefebvre, 2013). Many view marketing as a manipulative practice (Spotswood et al., 2012) that encourages consumption linked to inequalities (Andreasen, 2006) and environmental degradation (Hastings, 2007). As with social media confusion, this is a deep-rooted and pervasive issue. Social marketing has had and continues to have a complicated relationship with commercial marketing (Spotswood et al., 2012). Further, many fail to differentiate between marketing and advertising. It is necessary to acknowledge the relationship itself and its complexity due to its potential influence upon stakeholders, decision-makers, and policymakers. While social marketing has now been accepted as *"part of the marketing mainstream"* (Dibb & Carrigan, 2013, p.1377), views differ as to the degree to which mainstream marketing practices should be integrated into social marketing efforts (Hastings & Saren, 2003; Peattie & Peattie, 2003). As an example, the 4Ps concept, which is central to mainstream marketing but also subject to ongoing criticism within social marketing (Gordon, 2012), is not explicitly listed as a "core concept" of the discipline by the social marketing associations, while segmentation is included (ISMA et al., 2017).

### Social marketing identity

Strategies to drive change come in many forms, and social marketing as a discipline has been affected by a lack of clarity around its own "label". Many people mistakenly equate social advertising, strategic communications, and educational outreach with social marketing, calling themselves "social marketers" when they should not be doing so (Donovan, 2011; French & Gordon, 2015; Spotswood & Tapp, 2013). The inverse situation also exists, where people who are legitimately using a social marketing conceptual framework do not call themselves social marketers or refer to the work as social marketing (Kassirer et al., 2019). This may be a conscious decision adopted to avoid the confusion and misperceptions about social marketing, or it may be unintentional due to a lack of awareness about social marketing as a discipline. Deshpande (2019) has proposed categorising social change managers based on social marketing benchmarks and determining who social marketing managers are and who are not.

The benchmark criteria, developed by Andreasen (2002) and revised by others (French & Blair-Stevens, 2006; French & Russell-Bennett, 2015; Xia et al., 2016), arguably remains the standard by which social marketing programmes are defined and evaluated. The recently developed consensus on six social marketing concepts and an overarching principle, "*the facilitation of personal and social good*" (ISMA, AASM, & ESMA, 2017, p.2), provide a solid foundation for social marketing's identity and planning process. The discipline has also developed a statement of ethics (Kubacki et al., 2020), strengthening social marketing's identity by improving practice. Given the availability of this collective guidance, social marketing scholars have made the explicit request to other academics and practitioners to appropriately label themselves and their work (Deshpande, 2019; Kassirer et al., 2019). French and Gordon (2015, p.34) have warned that a failure to address erroneous views of social marketing *"could lead to its marginalisation"*.

One way to address social marketing's identity issues and to demonstrate the effectiveness of social marketing strategies is to undertake a comprehensive effort to compile and share the discipline's extensive evidence base and explicitly attribute these success stories to the discipline (Deshpande, 2019; French & Evans, 2018; French & Gordon, 2015; Lefebvre, 2013; Rundle-Thiele et al., 2019). Relatedly, and at a broader scale, many have called for the discipline of social marketing to improve its marketing of itself (Andreasen, 2002; Beall et al., 2012; Wood, 2012). Others highlight the need to promote and protect the brand (Kassirer et al., 2019); suggestions include developing a stronger brand identity through words and visuals (Lee, 2020) and consistent or standardised language when describing the discipline. These discussions have served as a good starting point, but we believe that more robust engagement and debate is necessary at all levels within both academic and practitioner settings. This is necessary to avoid a reduction in social marketing's impact and effectiveness due to well-meaning yet misattributed efforts.

### Social marketing theory

Another early critique that persists today revolves around the use of theory in social marketing. Social marketing itself is not a theory; it is a sub-discipline of marketing and a structural guiding framework (Kotler & Zaltman, 1971; Lee & Kotler, 2019; Stead et al., 2007), drawing theory from multiple disciplines. Going back to the early 1980s, Bloom and Novelli (1981) noted that many social marketing studies lacked proper design and implementation, responding with a call for the discipline to develop a greater theoretical foundation; fifteen years later, (Lefebvre, 1996) repeated the same observation and recommendation. Despite a proliferation of the available theory, frameworks, and models (Akbar et al., 2019; French & Gordon, 2015), scholars still emphasise the under-utilisation of theory in social marketing; the following quote illustrates this sentiment: *"there is little guidance on which theory to use, for what purpose, and importantly there is limited guidance directing researchers on how to apply theory to deliver behavioural change"* (Rundle-Thiele et al., 2019, p.4).

Others have noted that many social marketers fail to use theory altogether (Kubacki & Rundle-Thiele, 2013; Truong, 2014) or default to familiar theories that may not apply in the particular context (Manikam & Russell-Bennett, 2016). Reassuringly, Lefebvre (2013), in turn citing numerous others (Blair-Stevens et al., 2010; Donovan & Henley, 2010; Hastings, 2007; Lefebvre, 2000; Novelli, 1990). While this is a known issue, increasing attention has been given to addressing it: *"many social marketers stress the need to base programs on empirically validated models and theories in order to understand and influence the many variables that affect human behaviour"* (Lefebvre, 2013, p.77).

Further, the new theoretically-based and holistic approaches such as critical social marketing (Gordon, 2011), macro-social marketing (Kennedy, 2017), and systems social marketing (Domegan et al., 2016; Flaherty et al., 2020) suggest that advancements are being made on this front, although social marketing has failed to produce theories unique to itself (Peattie & Peattie, 2003; Rundle-Thiele et al., 2019). The diversity of guiding frameworks that support social marketing practice, coupled with the inter-and multidisciplinary nature of the discipline, has ironically hindered developing an overarching and unifying theory. We argue that since social marketing is such a broad discipline and has undeniably matured, a unifying theory is now becoming necessary. Thus, social marketing draws from a diverse body of theory that may strengthen its application and effectiveness while at the same time hinder its cohesive development as a discipline.

### Social marketing academic courses

Another challenge social marketing faces is the lack of a sufficient number of formal academic courses and social marketing programmes (Kassirer et al., 2019; Lee, 2020). While academic courses in social marketing are available (Deshpande, 2019; Kelly, 2009, 2013), they are still relatively few. The majority are offered in North America and Global North countries, and they are concentrated within public health, business, and communications. As observed, social marketing does not appear to have a specific "academic home" despite an increase in social marketing–focused graduate theses (Truong & Dietrich, 2018) and dissertations (Truong et al., 2014). Truong and Dietrich (2018, p.69) asserted that *"if social marketing is to continue developing as an academic discipline, it is important that more academic programmes are created at the undergraduate and graduate levels so that future social marketers can be trained".* This view was recently echoed by a coalition of leading social marketing scholars and practitioners (Kassirer et al., 2019). We argue that an increase in academic coursework featuring social marketing would play a key role in addressing the challenges. Thus, there is both a need and a desire for more social marketing formal education opportunities.

### Social marketing vs behavioural economics

It is informative to compare the history of social marketing's acceptance to that of behavioural economics. It has been recommended that social marketers should look to the history of behavioural economics for guidance on advancing the discipline (Lotenberg, 2015). Behavioural economics is arguably more well-known than social marketing and appears to have more support at the governmental policy level worldwide (Dessart & van Bavel, 2017). Behavioural economics seems to have done a better job of positioning and marketing itself than social marketing. As a result, the sheer number of "behavioural insights" units and teams becoming established worldwide in government and the private sector (Wendel, 2020) attests to the increasing popularity and meteoric rise of behavioural economics. In a noteworthy development, behavioural economics reportedly supplanted social marketing at a governmental level in the UK (Gordon et al., 2016). Social marketing and behavioural economics are complementary strategies, both integrating much of the same behavioural science theory as foundations to their frameworks. Social marketing, however, is broader and arguably more versatile (Deshpande, 2019; French, 2011; Kotler, 2011; Lee & Kotler, 2019) and is differentiated by the integration of marketing theory and practice. As a discipline, behavioural economics is bolstered by numerous accounts in the popular literature of the people behind the discipline and their personal stories, along with the history of the underlying social science and cognitive psychological theory (Ariely, 2008; Kahneman, 2011; Levitt & Dubner, 2006; Lewis, 2017; Sunstein, 2019; Thaler, 2015; Thaler & Sunstein, 2009). These engaging accounts have made this body of theory accessible and exciting while explicitly identifying it with behavioural economics. However, we are unaware of popular writings that focus on social marketing in the same manner, and we argue that this has actively served as a liability to the discipline.

The challenges mentioned in this section are wide-ranging and pervasive, as evidenced by their continued presence in the literature. Therefore, we explore the issues faced by social marketing more thoroughly from the perspectives of social marketing academics and practitioners alongside opportunities to address the problems identified.

## Method

Our qualitative study solicits information from social marketing academics and practitioners. The sample was selected through the purposive sampling technique (Robinson, 2014) using specific recruitment criteria:

- A minimum of five years of experience in social marketing as an academic or practitioner
- Involvement (designing, planning and implementation) in a minimum of one social marketing programme

A total of 60 social marketing experts were approached through social marketing/marketing conferences and events and were followed up via LinkedIn and email to invite them to participate in the study. Of the 60 approached, nine agreed to take part. This may be because only three months were budgeted for the data collection process. Many potential participants were not able to take part within the given timeframe because of prior personal and professional commitments; further, as we reached out to many well-established, high-level "thought leaders," these individuals arguably have less capacity to engage in additional scholarly activities over and above their current professional burden. While this is a small number, we argue that the saturation point was reached during these interviews (Guest et al., 2006), which mitigates the limitation of having a small sample. In addition, the participants collectively have 137 years of experience in hand, either as social marketing academics or practitioners or both and have more than 400 publications on social marketing, health promotion, and behaviour change (see Table 1 for participants' profiles). This highlights the selected sample size characteristics as broadly representative of the overall social marketing community, which has 1,200 globally scattered members (Lee, 2020).

**Table 1:** Participants’ profiles

Participants	Experience	Location
P1	Working in commercial marketing and health communication with more than five years of experience as an academic and practitioner in the discipline of social marketing	USA
P2	Thirty years of experience in social marketing as an academic and practitioner	USA
P3	More than seven years of experience as a social marketing practitioner	Belgium
P4	Full-time academic with twenty years of experience in social marketing	Australia
P5	Experience in health marketing communication and the use of emotions in social marketing interventions with a minimum of five years of experience in social marketing	UK
P6	Over twenty years of experience as an academic and practitioner in public health, public policy, governmental agencies, and national bodies	UK
P7	Nearly fifteen years of experience in social marketing as a practitioner with experience in the environmental discipline	USA
P8	Ten years of experience as a practitioner in social marketing, public health, immigration, and transport	UK
P9	Over twenty-five years of experience in social marketing/public health as an academic as well as practitioner	Israel

A qualitative approach was considered the most appropriate because it allowed capturing the information and in-depth understanding of the participants' views (Creswell, 2007) on current challenges and opportunities of social marketing. A semi-structured interview method was employed as a data collection technique—an authentic technique to deal with a complex research problem (Rubin & Rubin, 2005, 2012), offering an opportunity to gather rich data through individual narratives. All the interviews were conducted either using Skype or WhatsApp (audio/video). The average duration of the interviews was 40 min. Participants were asked to identify critical challenges social marketing faces based on their experience and offer solutions to overcome these challenges (see the interview guide provided in Table 2). To increase validity in the results and reduce bias, all interview transcripts were sent back to the participants for their review before the commencement of the data analysis process (Hagens et al., 2009). The data were analysed using the six-step approach of thematic analysis (Braun & Clarke, 2006). First, the research team familiarised themselves with the data by reading the transcripts to understand the texts. Thus, the data was manually organised in a meaningful and systematic way. Second, the team generated initial codes, and themes were allowed to surface from the data. Third, the transcripts were read in detail to draw out content aligned with the identified themes. Finally, the identified themes were grouped and reviewed for accuracy by more than one researcher to reduce the researcher bias before analysis and discussion.

**Table 2:** Interview guide

Topic	Questions	Probes
Knowledge/experience in the field of social marketing	An overview of your experience in social marketing, either academic or practitioner	A minimum of five years of experience in social marketing as an academic or practitioner Involvement in at least one social marketing programme
Challenges faced by social marketing	In your view, what are the main challenges for social marketing as a field?	Challenge is defined as a problematic situation that needs an urgent solution
Opportunities for social marketing to overcome challenges	In your view, are there opportunities for social marketing to overcome some of the challenges?	Opportunity is defined as a chance, especially one that offers some advantage. An opportunity can be a combination of favourable circumstances or situations

## Findings and discussion

Three themes emerged from the data and are discussed in this section.

### Poor branding (as an internal challenge for social marketing)

Andreasen (2002) considered the identity of social marketing discipline to be poor, and others noted that it had not improved some 17 years later (Kassirer et al., 2019). Our findings suggest that social marketing has a strong identity, as evidenced by the broad applicability and implementation of social marketing from upstream to downstream social issues (Cook et al., 2021). However, the interviewees strongly echoed the field's poor branding, with six of the nine raising this as a serious concern. P4 felt that: *"we are still confused with communication and social media in the western world. In the non-western world, social marketing gets confused with contraceptive marketing, which governments think that's all we can do, that social marketing can only promote condoms and pills, which is not true; social marketing can do much more than that".* P4 further suggests that those working in the discipline need to make an effort to explain to others that the profession exists by focusing on the branding aspect of the field. P6 agrees with this but notes that describing what social marketing can offer to policymakers in simple ways is a considerable challenge.

*"So I think one of the challenges for social marketing is to position itself as being, you know, probably the best-formulated set of processes we've got for designing successful programmes that tackle causal issues as well as individual issues and have a sustained population of impact. But describing that in simple ways that politicians and policymakers can buy into easily is I think the major challenge"* P6.

Evidence of previous success is, of course, required in all forms of marketing, and clients and policymakers will always want to see examples of what has worked well, supported by rigorous evaluation data. This is standard practice in commercial marketing when bidding for work (Escalas, 2012) that social marketing can embrace to good advantage.

P8 feels social marketing continues *"to be misunderstood as an approach*", noting that marketing is not well understood per se by those not involved in it and that this does not help the situation, perhaps alluding to the sometimes unhelpful perceptions of commercial marketing (Andreasen, 2006; Deshpande, 2019; Hastings & Domegan, 2014; Lefebvre, 2013). While most interviewees who spoke on this theme viewed the discipline's poor branding as a challenge, P9 viewed the issue more as an opportunity. P9 felt that the discipline is becoming better known and understood and that there is more funding available for social marketing. Recognising the potential difficulties with the term "social marketing", P9 noted that they no longer use the term, preferring "social impact marketing" and that other terms used to describe work in this discipline include "social change marketing" and "behaviour change marketing". P2 raised a similar issue, noting that the donor organisation may want to describe the work with a particular term. The agency managing the change, therefore, adopts that term to describe the work.

*"If USAID is gonna give me $30 million, I'm gonna call it whatever USAID calls it…And right now, USAID only uses the term' social marketing' for family planning programmes. For some reason, when they're doing the same kind of work in other health areas, it's called 'social, and behaviour change', and that term has changed a bit over the years. They used to call it 'social and behaviour change communication', and now they've dropped the word communication because a lot of people argue that communication is only one of many tools for changing behaviours"* P2.

This is in line with the views of Deshpande (2019) that "social marketing" is not understood widely enough by donor organisations, or the association with commercial marketing noted by P8 is distasteful, such that other descriptors are used instead. With so many descriptors available for the work done in this discipline, it is perhaps no wonder that social marketing's branding is not clear. If the discipline's very name is part of the challenge, other challenges are associated with either reclaiming the name or changing it (Lee, 2020). As P9 points out, there are many research centres, journals, training courses, and degree programmes that use the term' social marketing' and that many are emotionally attached to it as a result. Changing the name of the discipline, if that is the solution to this challenge, would not be easy and could lead to more confusion. P2 indicated that because of using different names, social marketing is missing out on good publicity, for example,

*"So if you try and figure out what the literature tells us about how to do social marketing well, you'll miss half of the good work that's out there because a lot of times, it's not even called social marketing. So I think as a field we do ourselves a disservice by using overlapping and different, kind of, confusing terms when we're all talking about the same thing".* P2

Several interviewees mentioned the confusion with social media as a major issue for the discipline. For example, P2 opines that a lot of the good work done by social marketers is not found in the literature because it is not called "social marketing", and the confusion with social media leads to internet search results returning information about social media platforms rather than social marketing interventions. P9 relates this confusion with social media to the need for the discipline to reclaim its name or redefine itself, stating that *"as social marketers […] we need to do a better job of marketing social marketing"*. Probably in common with many working in the discipline, P9 feels that *"when I tell people that I work in social marketing […] they think I'm talking about social media"*. P8 has similar views:

*"I suppose big challenges are that we continue to be misunderstood as an approach, and that's got worse, so social marketing is mixed up with social media all the time. So that's a challenge. I mean, marketing isn't understood as well, so it's still mixed up with advertising and communications work, so we're always up against it a little bit in that regard".* P8

Given the weaknesses identified by the informants of this study in promoting social marketing to date, practitioners, academics, and associations have a key role in advocating social marketing. The views of P2 support this:

*"because we're so bad at being clear, in terms of how we label the work that we do…building out our evidence base and using consistent terminology is something that I've been harping about for a long time".* P2

The findings suggest that social marketing needs to address the social marketing paradox. The paradox is that social marketing is not often very good at promoting social marketing. As poor branding is considered an internal challenge for the field, we call for social marketing associations to work together to develop and help members to articulate the benefits of applying social marketing in ways that non-specialists and sceptics can relate to. This work should include disentangling social marketing from the negative connotations of commercial marketing. At the same time, social marketing professionals need to clarify social marketing from other similar disciplines. The most significant difference is that social marketing is based on fundamental marketing principles and a proven behaviour change technique. We argue that while a complete rebrand may not be necessary or advisable, some strategic "tweaks" may be helpful to create a stronger brand through consistent use of visual and verbal components.

### Competition (as an external challenge for social marketing)

It is noted that behavioural economics has gained much ground in recent years, displacing social marketing at the UK's government level (French & Gordon, 2020; Gordon et al., 2016). Interviewees were divided on whether the success of competing disciplines such as behavioural economics should be seen as a challenge or an opportunity. P7 felt that the fact that other social and behavioural change disciplines were well thought of was a "good thing":

*"getting good press" was a good thing, as it increases interest in the area generally, noting that even if we're jumping on the coattails of behavioural economics or design thinking, great, you know?"* P7

P6 mentions, "*I don't view it as competition, but it can be viewed as competition for attention."* P4, conversely, believes that social marketing is *"losing out to behavioural economics, they get the money, they get the seat […], so we struggle with funding and the mind-space of policymakers"*. Consistent with the views of Deshpande (2019), P4 concludes by echoing P9's view that social marketing needs better promotion at the policy level to compete with behavioural economics, as those in behavioural economics appear to have been more effective at promoting and advancing their discipline.

As noted by P4, other competing disciplines include communication, behavioural psychology, and programmes based on concepts from the behavioural sciences. P4 does not view this as competition, however. The disciplines are different, although they may have similar ends in mind (Dessart & van Bavel, 2017). Governments may be persuaded, in P4's view, relatively easy to fund programmes based on behavioural psychology and behavioural science as it's *"easy to do, it doesn't involve some of the things you don't want to do or legislate […], and it can save you money pretty quickly"*. Social marketing is qualitatively different as it attempts to understand the issue, the people, and the behaviour thoroughly to develop sustainable programmes with a measurable impact over time (French, 2017). However, this can be a more complex message for policymakers and donors who may want to see quick results and on tight budgets, as suggested by P6:

*The impact that behavioural psychology and behavioural science are having on policymaking, because their sell to government is, "come, you can avoid having to legislate or just tell people stuff by applying some of this relatively straightforward simple technique drawn from behavioural psychology and behavioural economics, and we can save you loads of money!" So it's easy to do, it doesn't involve some of the things you don't want to do, or legislate, and just nag people and it can, you know, so it's all simple, easy, you know, easy job, let's just do it. And I think we're not saying that; we're saying something a bit more complex than that".* P6

P6 further elaborates on how social marketing is pitching to policymakers as compared to behavioural economics:

*"We're saying you need to put effort into really understanding the issue, really understanding the people, developing collaborative programmes that are sustainable where there are clear aims and objectives, and we can measure their impact over time. But that takes a bit more effort. So our challenge, I think, is to communicate that extra effort, in terms of engagement, and then the process you have to go through, is worth it".* P6

P7 argues that although the challenge from behavioural economics was significant a few years ago when it claimed the limelight with a Nobel prize and a successful book, it is fading now to be replaced by design thinking. There is a feeling among some interviewees, perhaps because they are social marketers and are inherently biased towards the discipline, that social marketing is superior to behavioural economics and other similar approaches. P6 urges social marketers to be more critical of behavioural economics as it is being *"mis-sold"* to *"naïve governments"* who have not yet concluded that it is a *"useful part of what we [social marketers] do"* and on its own does not tackle the fundamental underlying causal issues driving behaviour and maintains *"the power imbalance between elites"*, as it is a purely downstream approach.

Overall, our findings suggest that competition is an external challenge for social marketing. However, many participants believe that this competitive environment actually helps social marketing tackle complex social issues. We suggest that competition with other disciplines would be more balanced with a strong branding of social marketing. Social marketing branding is weak and struggles to be recognised alongside more favoured approaches such as behavioural economics. While a rebrand to remove the words "social" and "marketing" might solve some of these problems, it would likely create a host of other problems, not the least of which would be what the discipline should be called instead and how those working in the discipline, both academics and practitioners, would manage the transition. The plethora of other disciplines that also have behaviour change as their primary goal was considered a hindrance and a benefit by the interviewees. While it seems sensible for social marketing to absorb good ideas from other disciplines (as many disciplines do), this does not help social marketing establish and maintain its brand. One might ask whether this matters. If the end goal is to achieve behaviour change, does it matter whether this is achieved through social marketing, behavioural economics, behavioural psychology, or design theory? The difference articulated by interviewees was that social marketing is broader and more complex than other disciplines and offers more tools, seeking to understand and use research evidence rather than merely introducing a product, service, or nudge campaign. Therefore, strong branding and better marketing would add further competitiveness and strengthen the social marketing agenda of addressing wicked problems.

### Reach (as an internal and external challenge for social marketing)

The reach of social marketing emerged as another theme; six of the nine interviewees made significant points. P1 noted that there are big challenges relating to cultural forces partly associated with opportunities for the discipline. P1 felt there was a *"disconnect"* between the culture of social marketing experts and that of the target audiences of social marketing programmes and that commercial marketing was better able to make the connection. Understanding how people think and understand their language and culture is a clear opportunity for social marketing.

*"We should always be thinking about what marketing is doing and marketing, your commercial marketing because that's what social marketing's all about is… They do such a great job infiltrating communities, going into the street, nightclubs, bars. Somehow, why is it that Nike and tobacco, JUUL get into communities so well, but social marketing has a hard time getting into people's lives, and I think part of it has to do with just getting, understanding the culture, the culture of the street, the culture of the people, how people talk. How people act, and that's to me a big opportunity as well".* P1

Social marketing is often concerned with behaviour change that people do not want to make and often worry about sensitive issues (French, 2017). P5 felt that devising campaign messages that do not offend the target audience (or others) is particularly challenging in the age of *"movements"* on social media. The example P5 gave of an anti-obesity campaign that featured French fries in a cigarette packet caused a lot of offence. Messages that are difficult to convey are made more difficult, according to P5, because "*in some cases, it's cold hard facts that people find offensive, rather than […] the emotional aspects of the tone of the message"*. P5 strongly felt that while the plethora of health advice available online on various social media platforms can be hugely beneficial, there is also a lot of conflicting information. A social marketing approach could *"break through that noise and offer clear, concise, and simple information, which results in behaviour change, hopefully"* (P5).

We believe that social marketing could address issues related to cultures and sub-cultures by working directly with them and making a genuine effort to understand them and their settings to ensure that messages are culturally appropriate and do not offend. Lack of understanding about cultures and contexts could result from the difference in cultures of the target audience and those designing social marketing initiatives. This makes the issue of consumer research in social marketing more pressing. In addition, social marketing reach is vast and international, and social issues are now global rather than local, notably environmental and health issues. Interviewees agreed that social marketing's opportunity to make a real difference at all levels is there for the taking.

P6 felt that social marketing has been *"internalised"* by the UK government and is doing quite well but may not be filtered down to local levels. However, social marketing in some parts of the world is *"not even on the agenda"* (Kassirer et al., 2019, p.4). Consistent with the views of Lee and Kotler (2019), P6 reiterated the importance of understanding behaviours rather than relying on new products or services to solve problems without the support of a social marketing programme to promote the desired behaviour. While P8 noted that the design stage of all programmes and lack of funding were always a challenge, P9 felt more optimistic, noting that social marketing as a discipline has been good at absorbing ideas from other disciplines such as behavioural economics, anthropology, and design, taking on board what works to help create behaviour change. This is an interesting view considering that other interviewees considered other disciplines as competitors in terms of attention and funding and given that there has been a call for social marketing to establish its theoretical underpinning, rather than borrowing from other disciplines (Rundle-Thiele et al., 2019). However, we believe that the lack of a clear, unifying theory for the discipline should not be viewed as a weakness but rather a reflection of a complex, mature, and contested discipline. There is much contention and disagreement within the disciplines that make up the social and behavioural sciences. Therefore, social marketers should develop the theory and principles of best and ethical practice based on empirical studies and open discourse to have the more expansive reach necessary for dealing with complex social issues (French & Gordon, 2020).

We believe social marketing needs to advocate continuously for its place at the table of policy formulation and delivery to broaden the reach. There is a need for the international and regional social marketing associations to continue supporting social marketing by setting out what the discipline can offer in terms of effective, efficient, and ethical practices in creating social good. It can be done by publication, advocacy, knowledge sharing within the discipline and with other fields. Social marketing is not considered by the informants of this study to be a permeable discipline of study and application but underpinned by an increasingly well-articulated set of principles and concepts. There is a need for ongoing work to refine and promote these principles of good practice within social marketing and other study and application disciplines. It is clear from the findings that social marketing is a diverse discipline of research and application; it is not a pure discipline in the traditional sense such as mathematics, physics, or chemistry, but like most other applied disciplines (including behavioural science, sociology, and economics), its theoretical base is an amalgam drawn from a variety of other disciplines of study and disciplines. This is not necessarily a weakness; in fact, many would argue that in a more integrated world, such complex and systems-influenced approaches that cross disciplinary boundaries and seek to draw on all that is known are more helpful.

Finally, there was a call from P7 for experienced social marketers to step up to fill the gaps that will be left when the discipline's original thought leaders retire and leave the discipline.

*"I do feel as if we've got, you know, our thought leaders, XXXX down to XXXX and XXXX phase. Like, the 80, 70, 60-year-olds. That [these] thought leaders retire [at some point] and stuff like that and seminal people within the field, and I do see this interest in the young people, the 20, 30 somethings, but I am concerned that we have a little bit of a gap in the 40, the 50s"* P7.

P7 felt there was hope as more people are interested in social marketing. Perhaps, more social marketing associations, education, training and certification in social marketing would be useful for a broader reach of the discipline. For example, short courses, workshops and seminars to raise awareness of the discipline and how it uses proven marketing concepts to deliver messaging to achieve behaviour change for social good. The aim of social marketing to reach its fullest potential can be achieved through strong branding and self-promotion. It may be alleviated through the same means, i.e. improved promotion of social marketing and its ability to deal with social issues downstream, midstream, and upstream (Wood, 2016).

Interviewees, in general, agreed that different approaches could be viewed as complementary, but social marketing has more to offer (Deshpande, 2019; Lee, 2020; Lee & Kotler, 2019). Six of the nine interviewees felt there were plenty of opportunities for social marketing to make a difference, for example, environment, health, and reaching out to deprived sections of society, and that there were *"plenty of public sector challenges that would benefit from using social marketing"* (P8). Five interviewees gave specific examples of how social marketing could be used to change behaviour around current issues to stand out from its competition:

- P1 – as a social marketer working in very deprived areas, P1 could see a significant gap in reaching those on the street, sick, poor, and on drugs – social marketing could help these groups.
- P3 –climate change issues demand behaviour change at all levels. P3 also felt the rise of far-right parties in elections was a social problem that could be tackled with social marketing.
- P4 – social marketing is often equated with contraceptive social media marketing in the non-western world, yet the discipline has much more to offer and can be used to tackle many of society's issues.
- P5 – weight loss education to encourage behaviour change in eating and diet habits could benefit from a social marketing approach as there is so much conflicting advice and information available from so many different sources, so" people just don't know where to start".
- P6 – this interviewee used Bill Gates' launch of a new restroom and sewage processing process as an example of a great product idea that would benefit from a social marketing approach. The product itself is not the answer – it is changing people's behaviour to use the product.

These examples demonstrate the wide range of issues internationally that would benefit from a social marketing approach and are therefore clear opportunities for the discipline.

## Final thoughts—critique of social marketing

This analysis of expert views has shown that social marketing does not have a problem with its identity – indeed, those who work in it and who teach and research in the area are sure of the discipline's roots, capabilities, and reach. The discipline is proven to be a good technique for use in behaviour change programmes. Many examples in its relatively short history demonstrate its success in various settings in downstream, midstream and upstream configurations. The discipline has two academic journals devoted to it, and several national and international associations show that it is well established and well recognised.

While identity is not perceived as a problem for social marketing, branding is another matter, with the experts noting the persistence of confusion with social media and commercial marketing. Some funders compounded the problem to use alternative descriptors to avoid any potential negative connotations connected to commercial marketing. This challenge can be met by highlighting the techniques' identity and success and adopting a consistent approach to describing them to funders and the government. Part of the challenge is the multidisciplinary nature of the discipline and those who work in it. Reconciling the strengths of the disciplines on which social marketing draws, making a virtue of them, and building on them to create a stronger brand deliberately pitched as multidisciplinary would help the discipline be recognised and understood better upstream and help improve its reach. Rather than renaming and rebranding social marketing, which would present another set of thorny problems, we argue that improved communication of the brand from social marketing academics and practitioners is required.

While branding is an internal issue for the social marketing discipline, competition from similar disciplines is an external issue and is viewed by the experts as an opportunity as much as it is a challenge. However, no matter how it is viewed, the competition for recognising the discipline will be lessened if the discipline is better known, understood and communicated to those looking to fund and manage behaviour change programmes for social good.

Aside from the issues of branding and competition, other important issues raised by the expert interviewees related to a lack of reach for the discipline, especially upstream to policymakers. The knock-on effect of this is a lack of funding or the loss of programmes to other disciplines when bidding for work. Social marketers could also fail to empathise with their target audiences, gain their confidence and trust, and fail to consider unintended consequences of behaviour change programmes. However, this last problem is not confined to social marketing and affects all disciplines seeking to change public behaviours. With its well-established and well-tried toolkit of marketing tools, especially consumer and stakeholder research and continuous feedback mechanisms, social marketing is better placed than most disciplines to solve this problem.

The Covid-19 pandemic of 2020 to 2021 has proven with dire consequences the importance of social marketing programmes worldwide and the necessity for these to be well designed, ethically managed and sustainably funded for long-term success. Social marketing is well poised to address significant, complex, sensitive, and difficult national and global issues relating to physical and mental health, the environment, racial equity and equality, gender and disability issues. Relevant journals lead the way, with special issues on emancipation in the *Journal of Marketing Management*, racial equity in the *Social Marketing Quarterly*, and UN's sustainable development goals in the *Journal of Social Marketing*.

To conclude, this article has analysed and advanced the debates around the challenges and opportunities for social marketing discipline using insight from expert practitioners and academics with a track record in the area. Key issues of branding, competition and reach have been explored. Some suggestions are made about how the discipline can meet these challenges and take the opportunities offered to do social good using marketing techniques. This is a call to action to all those working in the discipline to consolidate and strengthen its identity to improve external branding and reach. The special issue on the 50th year of the discipline in the *Journal of Social Marketing* seems an appropriate time to do this.

## Limitations

This research is limited by being based on a small sample. However, the sample comprised experts in the discipline, and clear themes emerged, and data reached a saturation point. A follow-up study to include a much larger sample of academics and practitioners would help to assess additional themes. In addition, this line of inquiry could further benefit from a mixed-methods approach, integrating quantitative methods such as a survey which would allow for a larger population to be queried.

A further limitation is that the research findings are based on just one question, asking what, in the view of these experts, are the opportunities and challenges of social marketing. This single question necessarily leads to a relatively limited set of responses. Further research with a larger sample incorporating quantitative methods could be conducted; other questions could be used to probe for more detail and, in particular, to explore potential approaches for addressing the identified challenges using the Leximancer data analysis technique. The Leximancer data analysis method provides word frequency counts and co-occurrence counts of concepts present in the transcripts (Heath & Swabey, 2014) and may uncover other issues for the social marketing field. Future research could also examine the views of policymakers and other potential funders of social marketing programmes to discover the barriers to understanding and solutions to overcome in ways other than those outlined here.

## References

[CR1] Akbar, M. B., Foote, L., Soraghan, C., Millard, R., & Spotswood, F. (2021). *What Causes Social Marketing Programs to Fail? A Qualitative Study*, 1–18. 10.1177/15245004211010202

[CR2] Akbar MB, French J, Lawson A (2019). Critical review on social marketing planning approaches. Social Business.

[CR3] Andreasen, A. R. (1995). *Marketing social change: Changing behavior to promote health, social development, and the environment* (1st ed.). Jossey-Bass

[CR4] Andreasen AR (2002). Marketing social marketing in the social change marketplace. Journal of Public Policy & Marketing.

[CR5] Andreasen, A. R. (2006). *Social marketing in the 21st century*. Sage Publications

[CR6] Andreasen, A. R. (2010). Opportunities and challenges in social marketing. In J. N. Sheth, & N. K. Malhotra (Eds.), *Wiley international encyclopedia of marketing* (pp. 1–9). Wiley. 10.4324/9781315890005.

[CR7] Ariely, D. (2008). *Predictably irrational: The hidden forces that shape our decisions* (1st ed.). Harper

[CR8] Beall T, Wayman J, D’Agostino H, Liang A, Perellis C (2012). Social marketing at a critical turning point. Journal of Social Marketing.

[CR9] Blair-Stevens, C., Reynolds, L., & Christopoulos, A. (2010). Behavioural theory: Understanding the key influences on human behavior. In French, J., Blair-Stevens, C., McVey, D., & Merritt R. (Eds.), *Social marketing and public health: Theory and practice* (pp. 45–65). Oxford University Press

[CR10] Bloom, P., & Novelli, W. (1981). Problems and challenges in social marketing. *Journal of Marketing, 45*(2), 79–88 12280283

[CR11] Braun V, Clarke V (2006). Using thematic analysis in psychology. Qualitative Research in Psychology.

[CR12] Brennan, L., Previte, J., & Fry, M. L. (2016). Social marketing’s consumer myopia: Applying a behavioural ecological model to address wicked problems. *Journal of Social Marketing,**6*(3), 219–239. 10.1108/JSOCM-12-2015-0079

[CR13] Brychkov D, Domegan C (2017). Social marketing and systems science: Past, present and future. Journal of Social Marketing.

[CR14] Carman J (1973). On the universality of marketing. Journal of Contemporary Business.

[CR15] Churchman CW (1967). Wicked problems. Management Science.

[CR16] Cook J, Lynes J, Fries S (2021). Exploring Mistakes and Failures in Social Marketing: The Inside Story. Social Marketing Quarterly.

[CR17] Creswell, J. W. (2007). *Research Design: Qualitative*. SAGE Publications. 10.4135/9781849208956

[CR18] Deshpande, S. (2019). Social marketing’s strengths, weaknesses, opportunities, and threats (SWOT): A commentary. *Social Marketing Quarterly,**24*(4), 1–12. 10.1177/1524500419881770

[CR19] Dessart FJ, van Bavel R (2017). Two converging paths: Behavioural sciences and social marketing for better policies. Journal of Social Marketing.

[CR20] Dibb, S., & Carrigan, M. (2013). Social marketing transformed: Kotler, Polonsky and Hastings reflect on social marketing in a period of social change. *European Journal of Marketing*. 10.1108/EJM-05-2013-0248.

[CR21] Domegan C, McHugh P, Devaney M, Duane S, Hogan M, Broome BJ, Layton RA, Joyce J, Mazzonetto M, Piwowarczyk J (2016). Systems-thinking social marketing: Conceptual extensions and empirical investigations. Journal of Marketing Management.

[CR22] Donovan, R. (2011). Social marketing’s mythunderstandings. *Journal of Social Marketing,**1*(1), 8–16. 10.1108/20426761111104392

[CR23] Donovan, R., & Henley, N. (2010). Principles and practice of social marketing: An international perspective. In *Principles and Practice of Social Marketing: An International Perspective*. 10.1017/CBO9780511761751.

[CR24] Escalas, J. E. (2012). *[chapter x] Success Stories: How Marketing Managers Can Leverage the Psychology of Narratives*. *April*.

[CR25] Flaherty T, Domegan C, Duane S, Brychkov D, Anand M (2020). Systems Social Marketing and Macro-Social Marketing: A Systematic Review. Social Marketing Quarterly.

[CR26] French J (2011). Why nudging is not enough. Journal of Social Marketing.

[CR27] French, J. (2017). *Social marketing and public health: Theory and practice* (second). Oxford University Press

[CR28] French, J., & Blair-Stevens, C. (2006). Social marketing national benchmark criteria. UK National Social Marketing Centre; available: http://www.snh.org.uk/pdfs/sgp/A328466.pdf. Accessed 2 Jan 2021

[CR29] French, J., & Evans, D. (2018). *Compilation of social marketing evidence of effectiveness: Key references 2018* (Issue November).

[CR30] French, J., & Gordon, R. (2015). *Strategic social marketing*. Sage Publications

[CR31] French, J., & Gordon, R. (2020). *Strategic Social Marketing: For Behaviour and Social Change* (2nd ed.). SAGE Publications, Inc

[CR32] French J, Russell-Bennett R (2015). A hierarchical model of social marketing. Journal of Social Marketing.

[CR33] Gordon, R. (2011). Critical social marketing: Definition, application and domain. *Journal of Social Marketing, 1*(2), 82–99. 10.1108/20426761111141850

[CR34] Gordon R (2012). Re-thinking and re-tooling the social marketing mix. Australasian Marketing Journal.

[CR35] Gordon R (2013). Unlocking the potential of upstream social marketing. European Journal of Marketing.

[CR36] Gordon R, Russell-Bennett R, Lefebvre RC (2016). Social marketing: The state of play and brokering the way forward. Journal of Marketing Management.

[CR37] Guest G, Bunce A, Johnson L (2006). How many interviews are enough?: An experiment with data saturation and variability. Field Methods.

[CR38] Hagens V, Dobrow MJ, Chafe R (2009). Interviewee transcript review: Assessing the impact on qualitative research. BMC Medical Research Methodology.

[CR39] Hastings, G. (2007). *The Potential of Social Marketing: Why should the Devil have all the best tunes?* Elsevier

[CR40] Hastings, G., & Domegan, C. (2014). *Social marketing: From tunes to symphonies* (2nd ed.). Routledge

[CR41] Hastings G, Donovan RJ (2002). International initiatives: Introduction and overview. Social Marketing Quarterly.

[CR42] Hastings G, Saren M (2003). The critical contribution of social marketing: Theory and application. Marketing Theory.

[CR43] Heath, A., & Swabey, K. (2014). Content and leximancer analysis as methodological tools to study formal school grievances: *Joint AARE-NZARE 2014 Conference, Brisbane*, 1–15.

[CR44] ISMA, AASM, & ESMA. (2017). *Global consensus on social marketing principles, concepts and techniques*.

[CR45] Kahneman, D. (2011). *Thinking, fast and slow* (1st ed.). Farrar.

[CR46] Kassirer, J., Lefebvre, C., Morgan, W., Russell-Bennett, R., Gordon, R., French, J., Suggs, L. S., Lee, N., & Biroscak, B. J. (2019). Social marketing comes of age: A brief history of the community of practice, profession, and related associations, with recommendations for future growth. In *Social Marketing Quarterly, 25*(3), 209–225. 10.1177/1524500419866206

[CR47] Kelly KJ (2009). Social marketing education: The beat goes on. Social Marketing Quarterly.

[CR48] Kelly KJ (2013). Academic course offerings in social marketing: The beat continues. Social Marketing Quarterly.

[CR49] Kennedy, A. M., Kemper, J. A., & Parsons, A. G. (2018). Upstream social marketing strategy. *Journal of Social Marketing*, *8*, 258–279

[CR50] Kennedy, A. M., & Parsons, A. (2012). Macro-social marketing and social engineering: A systems approach. *Journal of Social Marketing,**2*(1), 37–51. 10.1108/20426761211203247

[CR51] Kennedy, A. M. (2017). Macro-Social Marketing Research: Philosophy, Methodology and Methods. *Journal of Macromarketing, 37*(4), 347–355. 10.1177/0276146717735467

[CR52] Kotler, P. (2011). Behavioral economics or social marketing? The latter! *The Sunday Times*.

[CR53] Kotler, P., & Zaltman, G. (1971). Social marketing: An approach to planned social change. *Journal of Marketing, 35*(3), 3–12 12276120

[CR54] Kubacki, K., Eagle, L., French, J., Mintz, J., Musumba, D., & Ib, P. (2020). *Social Marketing Statement of Ethics*. 10.5281/zenodo.4087987.

[CR55] Kubacki, K., & Rundle-Thiele, S. (2013). *Contemporary issues in social marketing*. Cambridge Scholars Publishing

[CR56] Lee, N. R. (2020). The future of social marketing: Let's get it in orbit by 2025! *Social Marketing Quarterly*, 1–11. 10.1177/1524500419889141.

[CR57] Lee, N. R., & Kotler, P. (2019). *Social marketing: Behavior change for social good* (6th ed.). SAGE Publications, Inc.

[CR58] Lefebvre, R. C. (1996). 25 years of Social Marketing: looking back to the future. *Social Marketing Quarterly, 3*(3–4), 51–58

[CR59] Lefebvre, R. C. (2000). Theories and models in social marketing. In P. N. Bloom, & G. T. Gundlach (Eds.), *Handbook of Marketing and Society*. SAGE Publications, Inc

[CR60] Lefebvre, R. C. (2013). *Social Marketing and Social Change: Strategies and Tools For Improving Health, Well-Being, and the Environment*. Jossey-Bass.

[CR61] Levitt, S. D., & Dubner, S. J. (2006). *Freakonomics: A rogue economist explores the hidden side of everything*. William Morrow.

[CR62] Lewis, M. (2017). *The undoing project: A friendship that changed our minds* (First edit). W.W. Norton & Company.

[CR63] Lotenberg LD (2015). What can social marketers learn from the accomplishments of behavioral economics?. Social Marketing Quarterly.

[CR64] Luck DJ (1969). Broadening the concept of marketing – too far. Journal of Marketing.

[CR65] Manikam S, Russell-Bennett R (2016). The social marketing theory-based (SMT) approach for designing interventions. Journal of Social Marketing.

[CR66] McDermott L, Stead M, Hastings G (2005). What is and what is not social marketing: The challenge of reviewing the evidence. Journal of Marketing Management.

[CR67] Novelli, W. D. (1990). Applying social marketing to health promotion and disease prevention. In K. Glanz, F. M. Lewis, & B. K. Rimer (Eds.), *Health behavior and health education: Theory, research, and practice* (pp. 342–369). Jossey-Bass.

[CR68] Patten ML, Patten ML (2018). Qualitative research design. Understanding Research Methods.

[CR69] Peattie, S., & Peattie, K. (2003). Ready to fly solo? Reducing social marketing’s dependence on commercial marketing theory. *Marketing Theory,**3*(3), 365–385. 10.1177/147059310333006

[CR70] Robinson RS (2014). Purposive sampling. Encyclopedia of Quality of Life and Well-Being Research.

[CR71] Rubin, H. J., & Rubin, I. S. (2005). Qualitative interviewing – The art of hearing data. (second ed.). SAGE Publications, Inc

[CR72] Rubin, H., & Rubin, I. (2012). The Responsive Interview as an Extended Conversation. In *Qualitative Interviewing (2nd ed.): The Art of Hearing Data*. 10.4135/9781452226651.n6.

[CR73] Rundle-Thiele S, David P, Willmott T, Pang B, Eagle L, Hay R (2019). Social marketing theory development goals: An agenda to drive change. Journal of Marketing Management.

[CR74] Spotswood F, Tapp A (2013). Beyond persuasion: A cultural perspective of behaviour. Journal of Social Marketing.

[CR75] Spotswood F, Tapp A, French J, Stead M (2012). Some reasonable but uncomfortable questions about social marketing. Journal of Social Marketing.

[CR76] Stead M, Gordon R, Angus K, McDermott L (2007). A systematic review of social marketing effectiveness. Health Education.

[CR77] Sunstein, C. R. (2019). *How change happens*. MIT Press.

[CR78] Thaler, R. H. (2015). *Misbehaving: The making of behavioral economics*. W.W. Norton & Company.

[CR79] Thaler, R. H., & Sunstein, C. R. (2009). *Nudge: Improving decisions about health, wealth, and happiness*. Penguin Books.

[CR80] Trenchard-Mabere, E. (2016). The emergence of systems thinking in behaviour change: A public health focus. In Fiona Spotswood (Ed.), *Beyond Behaviour Change* (pp. 257–281). Policy Press.

[CR81] Truong VD (2014). Social marketing: A systematic review of research 1998–2012. Social Marketing Quarterly.

[CR82] Truong, V. D., & Dietrich, T. (2018). Master’s thesis research in social marketing (1971–2015). *Journal of Social Marketing,**8*(1), 58–98.

[CR83] Truong VD, Hall CM, Garry T (2014). Social marketing as the subject of doctoral dissertations. Social Marketing Quarterly.

[CR84] Truong VD, Saunders SG, Dong XD (2019). Systems social marketing: A critical appraisal. Journal of Social Marketing.

[CR85] Veríssimo D (2020). Taking the pulse of social marketing: The 2019 world social marketing conference. Social Marketing Quarterly.

[CR86] Wendel, S. (2020). *Designing for Behavior Change: Applying Psychology and Behavioral Economics* (2nd ed.). O'Reilly Media, Inc.

[CR87] Wood M (2012). Marketing social marketing. Journal of Social Marketing.

[CR88] Wood M (2016). Midstream social marketing and the co-creation of public services. Journal of Social Marketing.

[CR89] Xia Y, Deshpande S, Bonates T (2016). Effectiveness of social marketing interventions to promote physical activity among adults: A systematic review. Journal of Physical Activity and Health.

